# Noninvasive assessment of pulmonary arterial capacitance by pulmonary annular motion velocity in children with ventricular septal defect

**DOI:** 10.1186/s12947-016-0081-4

**Published:** 2016-09-07

**Authors:** Yasunobu Hayabuchi, Akemi Ono, Yukako Homma, Shoji Kagami

**Affiliations:** Department of Pediatrics, Tokushima University, Kuramoto-cho-3, Tokushima, 770-8305 Japan

**Keywords:** Tissue Doppler imaging, Pulmonary annular motion, Pulmonary arterial compliance, Children

## Abstract

**Background:**

We hypothesized that longitudinal pulmonary arterial deformation during the cardiac cycle reflects pulmonary arterial capacitance. To examine this hypothesis, we assessed whether tissue Doppler-derived pulmonary annular motion could serve as a novel way to evaluate pulmonary arterial capacitance in pediatric patients with ventricular septal defect (VSD).

**Methods:**

In this prospective study, pulmonary annular velocity was measured in children (age, 6 months–5 years) with a preoperative VSD (VSD group, *n* = 35) and age-matched healthy children (Control group, *n* = 23). Pulmonary artery capacitance was calculated by two methods. Systolic pulmonary arterial capacitance (sPAC) was expressed as the stroke volume/pulmonary arterial pulse pressure. Diastolic pulmonary arterial capacitance (dPAC) was determined according to a two-element windkessel model of the pulmonary arterial diastolic pressure profile.

**Results:**

Pulmonary annular velocity waveforms comprised systolic bimodal (s1′ and s2′) and diastolic e’ and a’ waves in all participants. The peak velocities of s1′, s2′, and e’ were significantly lower in the VSD group than in the Control group. On multiple regression analysis, sPAC was an independent variable affecting the peak velocities of the s1′, s2′, and e’ waves (β = 0.41, 0.62, and 0.35, respectively). The dPAC affected the s1′ wave peak velocity (β = 0.34). The time durations of the s1′ and e’ waves were independently determined by the sPAC (β = 0.49 and 0.27).

**Conclusion:**

Pulmonary annular motion velocity evaluated using tissue Doppler is a promising method of assessing pulmonary arterial capacitance in children with VSD.

**Electronic supplementary material:**

The online version of this article (doi:10.1186/s12947-016-0081-4) contains supplementary material, which is available to authorized users.

## Background

Pulmonary vascular hemodynamic assessment has traditionally evaluated pulmonary vascular resistance (PVR) [1–3]. However, PVR primarily reflects small vessel status and the static component of the pulmonary circulation [4], and it does not reflect the properties of the large and medium pulmonary vessels or account for pulsatile elements of the pulmonary circulation. Pulmonary artery (PA) capacitance reflects the dynamic component of the pulmonary circulation and is thought to be determined largely by the properties of the large proximal capacitance vessels [5]. Because it is the immediate environment encountered by the right ventricle (RV), PA capacitance is a major determinant of RV work [5]. It has recently been shown that lower PA capacitance is strongly associated with worse survival in patients with pulmonary arterial hypertension (PAH) [5–7]. Furthermore, PA capacitance is also reported to be a strong prognostic indicator in patients with left ventricular dysfunction, even in patients with normal PVR [8]. Consequently, accurate determination and serial follow-up of PA capacitance are important in the management of patients with various cardiac diseases with congestive heart failure. However, despite its potential importance for RV performance and clinical outcomes, PA capacitance is not routinely measured. Noninvasive quantitative assessment of PA capacitance remains challenging.

We have previously reported that the pulmonary annular motion velocity waveform obtained using tissue Doppler imaging (TDI) reflects right ventricular outflow tract (RVOT) performance [9]. In the present study, we hypothesized that the pulmonary annular motion would be affected not only by RVOT function, but also by PA capacitance because the pulmonary annulus is located adjacent to the RVOT and PA. In large arteries, deformation of the arterial wall during systole and diastole occurs in the radial and longitudinal directions (Additional file 1). The present study attempted to assess the relationship between the longitudinal pulmonary arterial deformation and capacitance.

The aim of this study, therefore, was to determine whether tissue Doppler-derived pulmonary annular motion velocity can be used as a noninvasive assessment of PA capacitance in children with ventricular septal defect (VSD).

## Methods

### Study population

The prospective study group comprised consecutive 35 pediatric patients with a preoperative VSD (VSD group; mean age, 2.3 ± 1.5 years; range, 0.6–5.0 years). These patients were scheduled for surgical closure. Twenty-three age-matched healthy children without electrocardiographic or echocardiographic abnormalities (Control group; age, 2.5 ± 1.6 years; range, 0.6–5.0 years) were also enrolled. The patients underwent cardiac catheterization within 3 days of assessment by echocardiography. Data collected between December 2012 and December 2015 were analyzed. All protocols were approved by the Institutional Review Board of the Tokushima University Hospital and conformed to the ethical guidelines of the Declaration of Helsinki (1975). The parents of all subjects provided their written, informed consent for their children to participate in the study.

### Echocardiographic study

**Fig. 1:**
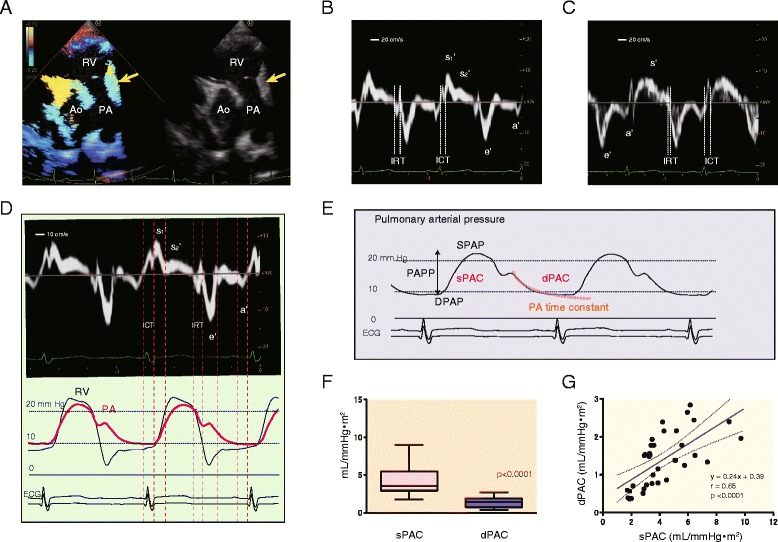
Recording of pulmonary annular motion velocity and measurement of pulmonary arterial capacitance. A representative recording of pulmonary annular motion evaluated by tissue Doppler imaging in a healthy 2-year-old boy is shown. The long-axis view of the right ventricular outflow tract and main pulmonary artery (PA) is visualized, and the sample volume is positioned on the RV free wall side of the pulmonary annulus, as indicated by the *yellow arrow* (**a**). Pulmonary annular velocity of the RV free wall side is determined (**b**). The tricuspid annular motion waveform from the same individual is also evaluated for the sake of comparison (**c**). The tissue Doppler-derived annular velocity waveform comprises s1′, s2′, e’, and a’ for the pulmonary annulus, and s’, e’, and a’ for the tricuspid annulus. Simultaneous recordings of pulmonary annular motion and RV and PA pressure curves in a 4-year-old girl with a ventricular septal defect (VSD) are shown (**d**). The measurements of systolic pulmonary arterial capacitance (sPAC) and diastolic pulmonary arterial capacitance (dPAC) are shown (**e**). The calculations to obtain sPAC and dPAC are described in the methods section. The comparison between sPAC and dPAC is shown in panel **f**. *Boxes* show the distribution (25th and 75th percentiles; central line, median). *Vertical lines* represent the range between the 5th and 95th percentiles. The relationship between sPAC and dPAC is shown in panel **g**. Ao, aorta; PA, pulmonary artery; RV, right ventricle; ICT, isovolumic contraction time; IRT, isovolumic relaxation time; sPAC, systolic pulmonary arterial capacitance; dPAC, diastolic pulmonary arterial capacitance; SPAP, systolic pulmonary arterial pressure; DPAP, diastolic pulmonary arterial pressure; PAPP, pulmonary arterial pulse pressure

Standard and pulsed Doppler tissue echocardiography proceeded using a Preirus digital ultrasound system (Hitachi-Aloka Medical Co., Tokyo, Japan) equipped with 1–5 and 3–7 MHz sector transducers. All Doppler data were acquired from patients in the left lateral decubitus position during shallow respiration or end-expiratory apnea. Pulmonary annular motion velocity was measured using TDI in the long-axis view of the RVOT and PA. Guided by the two-dimensional images, a sample volume with a fixed length of 5.0 mm was placed on the pulmonary annulus of the RV free wall side, as indicated by the yellow arrow [9]. Figures 1a and b show a representative example of the color TDI and profile of the pulmonary annular velocity in a healthy child. Furthermore, tricuspid annular motion was recorded in the four-chamber view for the sake of comparison (Fig. 1c). The ultrasound beam was positioned parallel to the direction of the pulmonary and tricuspid annular motions. All tissue Doppler parameters were measured during three consecutive heart cycles by a single physician who was blinded to patient condition, and mean values were calculated.

In addition to pulsed TDI, participants were assessed by conventional, two-dimensional, M-mode, pulsed, continuous, and color Doppler echocardiography. The left ventricular ejection fraction (LVEF) was calculated from apical two-chamber and four-chamber images using the biplane Simpson’s technique. All parameters were measured over three cardiac cycles and then averaged.

### Cardiac catheterization

All VSD patients underwent cardiac catheterization within 3 days of echocardiography. Catheterization and angiography using an Integris Allura 9 Biplane (Phillips Medical Systems, Best, The Netherlands) proceeded using 4–6 Fr catheters. All patients were intubated and examined by biplane anteroposterior and lateral projection angiography. Ventricular volume was assessed by means of ventriculography and calculated using Simpson’s rule by quantitative CAW2000 cardiac analysis software (ELK Corporation, Osaka, Japan). During cardiac catheterization, the main PA pressure was measured using a high-fidelity manometer-tipped 0.014-inch pressure wire (PressureWire Aeris; St Jude Medical, Inc., St. Paul, MN, USA) to compare with pulmonary annular motion velocity (Fig. 1d). Pulmonary blood flow was calculated using the Fick principle. Stroke volume indexed to body surface area (BSA) was calculated from the pulmonary blood flow in 1 min divided by the heart rate (HR), expressed in mL/m^2^. PVR was calculated using the standard formula; that is, PVR was calculated as the mean PAP minus PA wedge pressure/pulmonary blood flow (PVR; expressed in Wood units∙m^2^).

PA capacitance was calculated by two methods. One was designated systolic PA capacitance (sPAC), which can be determined from measures of pulse pressure and stroke volume indexed to BSA. The sPAC was expressed as the stroke volume/pulse pressure (sPAC; mL/mmHg · m^2^) [6–8]. Furthermore, PA capacitance was also estimated in a different way. Diastolic PA capacitance (dPAC) was approximated according to a two-element windkessel model that assumes that compliance and hemodynamic resistance are constant during the measurement [10–12]. Previous studies reported that PA capacitance can be calculated from the PA pressure profile during diastole. The time constant (tau) of the exponentially decaying curve can be obtained from the fitting curve calculated from diastolic PA pressure. The dPAC (mL/mmHg · m^2^) was calculated from PVR and the PA time constant (tau) (Fig. 1e).

### Statistical analysis

All data are expressed as means ± standard deviation (S.D.) or as medians with the 5th–95th percentiles. Statistical significance was determined using the Mann-Whitney *U*-test or Student’s *t*-test, as appropriate. Linear regression analyses were performed for correlations between the pulmonary annular motion velocity and hemodynamic parameters, and Pearson’s or Spearman’s correlation coefficients were calculated, as appropriate. Variables with *p* < 0.10 on univariate analysis were “candidates” for a backwards stepwise (inclusion criteria/exclusion criteria: *p* < 0.05/*p* > 0.1, respectively) multivariate analysis, and those with *p* < 0.05 were “retained” in the model. Multiple regression analysis was used to identify hemodynamic variables affecting the pulmonary annular motion waveform. All statistical data were calculated using Prism version 6.0 (GraphPad Software, San Diego, CA, USA) and JMP 11 (SAS Institute, Inc., Cary, NC, USA) installed on a desktop computer. A value of *p* < 0.05 was considered significant. Intra-observer and inter-observer reproducibilities of TDI measurements were assessed using Bland-Altman analysis in a blinded manner. Data were recorded and assessed at 5-minute intervals by observers 1 and 2 from 20 randomly selected participants (VSD, *n* = 10; Controls, *n* = 10). For intra-observer variability, data were analyzed twice, 8 weeks apart. Inter-observer variability was assessed by analyzing data from two separate observers blinded to each other’s results.

## Results

No subjects were excluded from the subsequent analyses due to suboptimal recording from poor echocardiographic imaging. Accordingly, the study group included 23 healthy children (2.5 ± 1.6 years; range, 0.6–5.0 years) and 35 VSD patients (mean age, 2.3 ± 1.5 years; range, 0.6–5.0 years). Table 1 shows the clinical, echocardiographic, and hemodynamic data of the participants. Age, height, and HR did not differ significantly between the VSD group and the Control group, whereas body weight and BSA were significantly lower in the VSD group than in the Control group. Left ventricular end-diastolic dimension (LVEDD), left ventricular fractional shortening (LVFS), and LVEF were significantly higher in the VSD group than in the Control group.

**Table 1:** Clinical characteristics of the subjects

		Control (*n* = 23)	VSD (*n* = 35)	*p* values
Sex (male/female)		11/12	20/15	n.s.
Age (y)		2.5 ± 1.6	2.3 ± 1.5	n.s.
Weight (kg)		13.8 ± 3.3	11.4 ± 4.1	0.0224
Height (cm)		91.1 ± 7.1	88.7 ± 8.1	n.s.
Body surface area (m^2^)		0.58 ± 0.07	0.53 ± 0.08	0.0177
Heart rate (bpm)		90 ± 12	92 ± 17	n.s.
QRS duration (msec)		87 ± 6	89 ± 13	n.s.
Systolic blood pressure (mmHg)		80 ± 7	77 ± 6	n.s.
Diastolic blood pressure (mmHg)		46 ± 6	45 ± 6	n.s.
LVEDD (mm)		25.8 ± 3.1	29.2 ± 4.4	0.0022
LVFS (%)		32.6 ± 5.9	38.7 ± 6.1	0.0004
LVEF (%)		63.4 ± 5.6	69.3 ± 6.2	0.0005
Qp/Qs		-	2.04 ± 0.92	-
RVSP (mmHg)		-	35.4 ± 13.0	-
RVEDP (mmHg)		-	7.6 ± 2.1	-
RVEF (%)		-	53.0 ± 9.1	-
SPAP (mmHg)		-	29.0 ± 9.9	-
DPAP (mmHg)		-	11.5 ± 3.3	-
MPAP (mmHg)		-	19.7 ± 6.2	-
PAPP (mmHg)		-	17.5 ± 8.0	-
PVRi (Wood U∙m^2^)		-	1.44 ± 0.8	-
PASV (ml/m^2^)		-	62.7 ± 20.7	-
Transmitral flow (m/sec)	E	1.05 ± 0.17	1.11 ± 0.19	n.s.
	A	0.41 ± 0.07	0.51 ± 0.17	0.0099
Transtricuspid flow (m/sec)	E	0.64 ± 0.12	0.49 ± 0.18	0.0009
	A	0.29 ± 0.09	0.31 ± 0.13	n.s.
Mitral annular motion (cm/sec)	s’	8.4 ± 1.6	9.5 ± 2.7	0.0292
	e’	14.6 ± 2.8	15.7 ± 2.9	n.s.
	a’	5.0 ± 2.3	6.1 ± 2.4	n.s.
Tricuspid annular motion (cm/sec)	s’	12.5 ± 2.2	13.6 ± 1.8	0.0418
	e’	14.2 ± 2.3	12.7 ± 3.2	n.s.
	a’	6.6 ± 2.3	6.5 ± 2.1	n.s.

*LVEDD* left ventricular end-diastolic dimension, *LVFS* left ventricular fractional shortening, *LVEF* left ventricular ejection fraction, *Qp/Qs* pulmonary to systemic blood flow ratio, *RVSP* right ventricular systolic pressure, *RVEDP* right ventricular end-diastolic pressure, *RVEF* right ventricular ejection fraction, *SPAP* systolic pulmonary arterial pressure, *DPAP* diastolic pulmonary arterial pressure, *MPAP* mean pulmonary arterial pressure, *PAPP* pulmonary arterial pulse pressure, *PVRi* pulmonary vascular resistance indexed for body surface area, *PASV* pulmonary artery stroke volume, *n.s.* not significant

Figure 1b shows the pulmonary annular velocity curve. The tricuspid annular motion is shown in Fig. 1c for comparison with the pulmonary annular velocity waveform. The systolic wave was monomodal (s’) for the tricuspid annular velocity and bimodal (s1′ and s2′) for the pulmonary annular motion velocity. The systolic waveform peaked earlier in pulmonary annular motion than in the tricuspid annular velocity curve. The shapes of the e’ and a’ waves in diastole were similar. Simultaneous recordings of pulmonary annular motion, RV pressure, and PA pressure in a patient with VSD are shown (Fig. 1d). The pulmonary s1′ wave corresponds to the steep RV pressure elevation during early systole. The s2′ wave coincides with mid to late systolic phase around the peak RV and PA pressure. Measurements of the sPAC and the dPAC are shown with the PA pressure profile (Fig. 1e). The comparison between the sPAC and dPAC obtained from the VSD group is demonstrated in Fig. 1f. The sPAC was significantly higher than the dPAC (4.2 ± 1.9 vs. 1.4 ± 0.7 mL/mmHg · m^2^, *p* < 0.0001). There was a significant correlation between these parameters (Fig. 1g; *r* = 0.65, *p* < 0.0001).

**Fig. 2:**
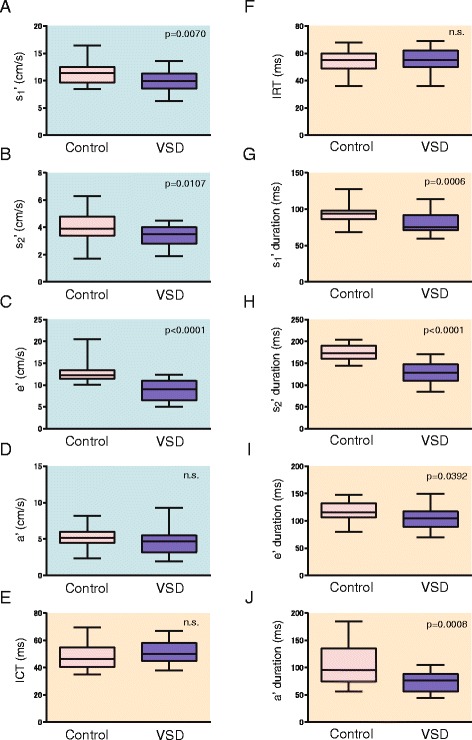
Comparison of pulmonary annular and tricuspid annular motion velocities between the Control group and the VSD group. The comparison is shown in terms of peak velocity (**a**–**d**) and time duration (**e**–**j**). *Boxes* show the distribution of peak velocity (25th and 75th percentiles; central line, median). *Vertical lines* represent the range between the 5th and 95th percentiles. ICT, isovolumic contraction time; IRT, isovolumic relaxation time

Pulmonary annular motion was compared between the Control and VSD groups (Fig. 2). The peak velocities of the s1′, s2′, and e’ waves were significantly lower in the VSD group than in the Control group (9.9 ± 1.9 vs. 11.5 ± 2.2 cm/s, *p* = 0.0070; 3.4 ± 0.7 vs. 4.1 ± 1.1 cm/s, *p* = 0.0107; 8.8 ± 2.3 vs. 13.0 ± 2.7 cm/s, *p* < 0.0001, respectively). The s1′ duration, s2′ duration, e’ duration, and a’ duration were significantly shorter in the VSD group (*p* = 0.0008, <0.0001, 0.0392, and 0.0008, respectively), whereas isovolumic contraction time (ICT) and isovolumic relaxation time (IRT) were not significantly different between the two groups.

**Fig. 3:**
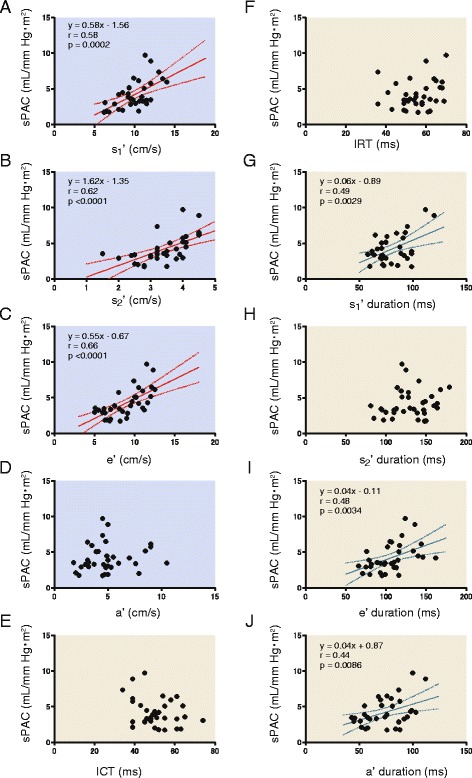
The relationship between the pulmonary annular motion waveform and systolic pulmonary arterial capacitance (sPAC) in the VSD group. The relationship was evaluated in terms of peak velocity (**a**–**d**) and time duration (**e**–**j**) in each wave. There are significant correlations between the peak velocities of pulmonary s1′, s2′, e’, and sPAC (**a**–**c**). The pulmonary s1′, e’, and a’ wave durations are significantly correlated with sPAC (**g**, **i**, and **j**, respectively). Linear regression lines with 95 % confidence interval (*dashed lines*) are indicated. sPAC, systolic pulmonary arterial capacitance; ICT, isovolumic contraction time; IRT, isovolumic relaxation time

**Fig. 4:**
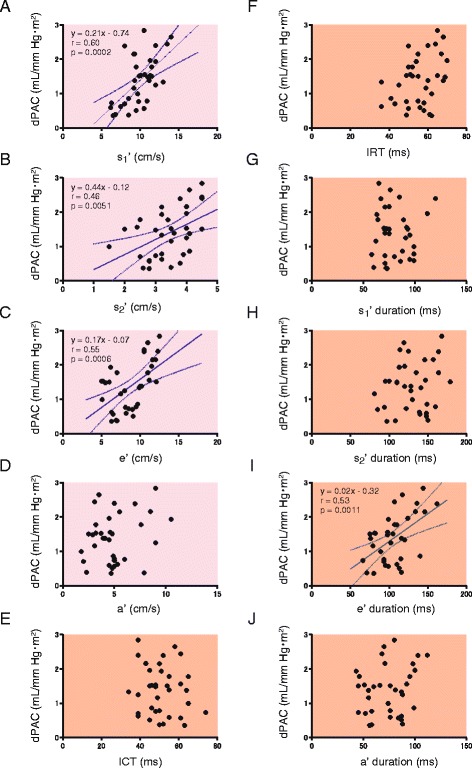
The relationship between the pulmonary annular motion waveform and diastolic pulmonary arterial capacitance (dPAC) in the VSD group. The relationship was evaluated in terms of peak velocity (**a**–**d**) and time duration (**e**–**j**) in each wave. There are significant correlations between the peak velocities of pulmonary s1′, s2′, e’ and dPAC (**a**–**c**). The e’ wave duration is significantly correlated with dPAC (I). Linear regression lines with 95 % confidence interval (*dashed lines*) are indicated. dPAC, diastolic pulmonary arterial capacitance; ICT, isovolumic contraction time; IRT, isovolumic relaxation time

Next, the relationships between the parameters obtained from TDI-derived pulmonary and tricuspid annular motion and right heart performance were examined in the VSD group. Table 2 summarizes the univariate regression analysis. TDI-derived parameters were significantly correlated with and determined by RV and PA function. Figure 3 shows the relationship between the sPAC and the pulmonary annular motion waveform. The peak velocity of the pulmonary annular s1′ wave was significantly correlated with the sPAC (*r* = 0.58, *p* = 0.0002). The s2′ and e’ velocity also showed significant correlations with the sPAC (*r* = 0.62, *p* <0.0001; *r* = 0.66, *p* <0.0001; respectively). ICT and IRT measured using TDI and the time duration of each wave were also assessed in terms of their correlations with the sPAC. The sPAC had significant correlations with s1′ and e’ and a’ wave duration (*r* = 0.49, *p* = 0.0029; *r* = 0.48, *p* = 0.0034; *r* = 0.44, *p* = 0.0088; respectively), whereas there were no significant correlations with ICT, IRT, and s2′ wave duration. Figure 4 shows the relationship between dPAC and TDI-derived pulmonary annular motion. The peak velocities of pulmonary annular s1′, s2′, and e’ wave were significantly correlated with the dPAC (*r* = 0.60, *p* = 0.0002; *r* = 0.46, *p* = 0.0051; *r* = 0.55, *p* = 0.0006; respectively). In regard to the timing issue, the e’ wave duration was significantly correlated with the dPAC (*r* = 0.53, *p* = 0.0011).

**Table 2:** Correlations between tissue Doppler parameters and pulmonary hemodynamics

	sPAC	dPAC	SPAP	DPAP	MPAP	PAPP	RVSP	RVEDP	PVRi	PASV	Qp/Qs	RVEF
Pulmonary annulus motion												
s1′ (cm/sec)	*r* = 0.58 *p* = 0.0002	*r* = 0.60 *p* = 0.0002	*r* = −0.39 *p* = 0.0209	n.s.	*r* = −0.45 *p* = 0.0072	*r* = −0.35 *p* = 0.0386	*r* = −0.41 *p* = 0.0138	*r* = −0.34 *p* = 0.0431	*r* = −0.59 *p* = 0.0002	n.s.	n.s.	*r* = 0.35 *p* = 0.0362
s2′ (cm/sec)	*r* = 0.62 *p* < 0.0001	*r* = 0.46 *p* = 0.0051	*r* = −0.50 *p* = 0.0022	n.s.	*r* = −0.41 *p* = 0.0141	*r* = −0.50 *p* = 0.0022	*r* = −0.45 *p* = 0.0071	n.s.	*r* = −0.43 *p* = 0.0104	n.s.	n.s.	n.s.
e’ (cm/sec)	*r* = 0.66 *p* < 0.0001	*r* = 0.55 *p* = 0.0006	*r* = −0.59 *p* = 0.0002	n.s.	*r* = −0.44 *p* = 0.0078	*r* = −0.65 *p* < 0.0001	*r* = −0.57 *p* = 0.0004	*r* = 0.44 *p* = 0.0079	*r* = −0.55 *p* = 0.0006	n.s.	*r* = −0.50 *p* = 0.0023	n.s.
a’ (cm/sec)	n.s.	n.s.	n.s.	n.s.	n.s.	n.s.	n.s.	n.s.	n.s.	n.s.	n.s.	n.s.
ICT (msec)	n.s.	n.s.	n.s.	n.s.	n.s.	n.s.	n.s.	n.s.	n.s.	n.s.	n.s.	n.s.
IRT (msec)	n.s.	n.s.	n.s.	n.s.	n.s.	n.s.	n.s.	n.s.	n.s.	n.s.	n.s.	n.s.
s1′ duration (msec)	*r* = 0.49 *p* = 0.0029	n.s.	n.s.	n.s.	n.s.	n.s.	n.s.	n.s.	n.s.	n.s.	n.s.	n.s.
s2′ duration (msec)	n.s.	n.s.	*r* = −0.42 *p* = 0.0128	n.s.	*r* = −0.35 *p* = 0.0378	*r* = −0.41 *p* = 0.0136	*r* = −0.35 *p* = 0.0394	n.s.	n.s.	n.s.	*r* = −0.47 *p* = 0.0047	n.s.
e’ duration (msec)	*r* = 0.48 *p* = 0.0034	*r* = 0.53 *p* = 0.0011	*r* = −0.67 *p* < 0.0001	*r* = −0.38 *p* = 0.0251	*r* = −0.58 *p* = 0.0003	*r* = −0.67 *p* < 0.0001	*r* = −0.58 *p* = 0.0003	*r* = 0.38 *p* = 0.0235	*r* = −0.46 *p* = 0.0056	n.s.	*r* = −0.63 *p* < 0.0001	n.s.
a’ duration	*r* = 0.44 *p* = 0.0086	n.s.	*r* = −0.53 *p* = 0.0011	n.s.	*r* = −0.47 *p* = 0.0046	*r* = −0.53 *p* = 0.0011	*r* = −0.47 *p* = 0.0044	*r* = 0.48 *p* = 0.0037	n.s.	n.s.	*r* = −0.64 *p* < 0.0001	n.s.
Tricuspid annulus motion												
s’ (cm/sec)	*r* = 0.36 *p* = 0.0332	*r* = 0.43 *p* = 0.0107	*r* = −0.47 *p* = 0.0043	n.s.	*r* = −0.39 *p* = 0.0193	*r* = −0.54 *p* = 0.0008	*r* = −0.35 *p* = 0.0418	n.s.	*r* = −0.38 *p* = 0.0260	n.s.	*r* = −0.53 *p* = 0.0011	*r* = 0.39 *p* = 0.0310
e’ (cm/sec)	n.s.	n.s.	*r* = −0.45 *p* = 0.0062	n.s.	n.s.	*r* = −0.41 *p* = 0.0136	*r* = −0.39 *p* = 0.0209	*r* = −0.34 *p* = 0.0451	n.s.	n.s.	*r* = −0.35 *p* = 0.0407	n.s.
a’ (cm/sec)	n.s.	n.s.	*r* = 0.53 *p* = 0.0011	*r* = 0.42 *p* = 0.0126	*r* = 0.49 *p* = 0.0028	*r* = 0.49 *p* = 0.0030	*r* = 0.49 *p* = 0.0024	n.s.	n.s.	n.s.	*r* = 0.55 *p* = 0.0006	n.s.
ICT (msec)	n.s.	n.s.	n.s.	n.s.	n.s.	n.s.	n.s.	n.s.	n.s.	n.s.	n.s.	n.s.
IRT (msec)	n.s.	n.s.	n.s.	n.s.	n.s.	n.s.	n.s.	n.s.	n.s.	n.s.	n.s.	n.s.
s’ duration (msec)	n.s.	n.s.	*r* = −0.41 *p* = 0.0118	n.s.	*r* = −0.39 *p* = 0.0228	*r* = −0.46 *p* = 0.0051	*r* = −0.43 *p* = 0.0021	n.s.	n.s.	n.s.	*r* = −0.36 *p* = 0.0138	n.s.
e’ duration (msec)	*r* = 0.35 *p* = 0.0232	n.s.	n.s.	n.s.	n.s.	n.s.	n.s.	n.s.	n.s.	n.s.	n.s.	n.s.
a’ duration (msec)	n.s.	n.s.	n.s.	n.s.	n.s.	n.s.	n.s.	n.s.	n.s.	n.s.	n.s.	n.s.

*sPAC* systolic pulmonary arterial capacitance, *dPAC* diastolic pulmonary arterial capacitance, *SPAP* systolic pulmonary arterial pressure, *DPAP* diastolic pulmonary arterial pressure, *MPAP* mean pulmonary arterial pressure, *PAPP* pulmonary arterial pulse pressure, *RVSP* right ventricular systolic pressure, *RVEDP* right ventricular end-diastolic pressure, *PVRi* pulmonary vascular resistance indexed for body surface area, *PASV* stroke volume to pulmonary artery, *Qp/Qs* pulmonary to systemic blood flow ratio, *RVEF* right ventricular ejection fraction

Next, multiple regression analysis for predictors of the pulmonary annular motion waveform was performed (Table 3). The analysis showed that the sPAC had an effect on the peak velocities of s1′, s2′, and the e’ wave and the time duration of the s1′ and a’ waves (β = 0.41, *p* = 0.0131; β = 0.62, *p* <0.0001; β = 0.35, *p* = 0.0314; β = 0.49, *p* = 0.0029; and β = 0.27, *p* = 0.0488, respectively). The dPAC affected the s1′ wave peak velocity (β = 0.34, *p* = 0.0354). The other right heart performance parameters, including RVEDP, RVSP, and Qp/Qs, also had an impact on pulmonary annular motion. Importantly, although RVEF had been assumed to determine pulmonary annular motion, no significant relationship was demonstrated with pulmonary annular motion in the VSD group.

**Table 3:** Results of stepwise multiple regression analysis for variables predicting accuracy of pulmonary annular motion parameters

Objective variable	Explanatory variables	B	SE	β	*p* values	VIF
s1′ (cm/sec)	sPAC	0.41	0.16	0.41	0.0131	1.76
	dPAC	0.95	0.43	0.34	0.0354	1.74
	RVEDP	−0.37	0.11	−0.39	0.0021	1.02
s2′ (cm/sec)	sPAC	0.24	0.05	0.62	<0.0001	1.00
e’ (cm/sec)	sPAC	0.37	0.19	0.35	0.0314	2.08
	RVSP	−0.05	0.02	−0.28	0.0432	1.71
	RVEDP	0.46	0.12	0.42	0.0005	1.04
a’ (cm/sec)	n.a.	-	-	-	-	-
ICT (msec)	n.a.	-	-	-	-	-
IRT (msec)	n.a.	-	-	-	-	-
s1′ duration (msec)	sPAC	3.76	1.17	0.49	0.0029	1.00
s2′ duration (msec)	Qp/Qs	−12.71	4.19	−0.47	0.0047	1.00
e’ duration (msec)	n.a.	-	-	-	-	-
a’ duration (msec)	sPAC	2.66	1.30	0.27	0.0488	1.11
	Qp/Qs	−9.19	3.18	−0.44	0.0070	1.48

*B* unstandardized coefficient, *SE* standard error, *β* standardized partial regression coefficient, *VIF* variance inflation factor, *sPAC* systolic pulmonary arterial capacitance, *dPAC* diastolic pulmonary arterial capacitance, *SPAP* systolic pulmonary arterial pressure, *RVSP* right ventricular systolic pressure, *RVEDP* right ventricular end-diastolic pressure, *Qp/Qs* pulmonary to systemic blood flow ratio, *n.a.* not available

### Reproducibility

The inter- and intra-observer reproducibilities of the TDI analysis of pulmonary annular motion were determined from Bland-Altman analysis of 20 randomly selected participants (VSD group, *n* = 10; Control group, *n* = 10). Table 4 shows the inter- and intra-observer reproducibilities obtained from the Bland-Altman plots (bias ± 2SDs [95 % limit of agreement]). They showed minimal bias and substantial agreement for reproducibility.

**Table 4:** Inter- and intra-observer reproducibility

Parameter variability	Inter-observer variability	Intra-observer
Peak velocity of s1′	0.15 ± 2.12 cm/s	−0.14 ± 2.14 cm/s
Peak velocity of s2′	−0.04 ± 1.25 cm/s	−0.05 ± 1.36 cm/s
Peak velocity of e’	0.27 ± 1.72 cm/s	0.29 ± 1.46 cm/s
Peak velocity pf a’	0.19 ± 1.43 cm/s	0.18 ± 2.41 cm/s
ICT	−5.34 ± 15.14 ms	−4.31 ± 10.22 ms
IRT	2.21 ± 10.03 ms	−1.31 ± 9.33 ms
s1′ duration	3.64 ± 10.31 ms	1.92 ± 8.13 ms
s2′ duration	−0.33 ± 18.75 ms	−0.29 ± 16.85 ms
e’ duration	0.88 ± 13.54 ms	−0.72 ± 11.14 ms
a’ duration	5.22 ± 9.36 ms	3.98 ± 6.38 ms

Inter- and intra-observer variabilities (bias ± 2 SD [95 % limit of agreement]) are shown

## Discussion

The present results showed that the pulmonary annular motion waveform is mainly determined by PA capacitance. Assessment of pulmonary annular motion is useful for estimating PA capacitance in children with VSD. Pulmonary annular TDI was found to be a simple, rapid, reproducible, and highly distinctive method for evaluating PA capacitance.

Recent reports demonstrated that PA capacitance has great impact on the prognosis in various cardiac diseases [5–8]. Patients require lifelong follow-up that includes serial assessment of PA capacitance. Therefore, noninvasive assessment of PA capacitance is considered quite important. However, its assessment is challenging due to the difficulty of measurement and poor reproducibility. Therefore, an invasive method is necessary to accurately assess PA capacitance in clinical practice. Several studies have been conducted to identify noninvasive ways for estimating PA capacitance using echocardiographic parameters [13, 14]. These studies attempted echocardiographic estimation of PA capacitance from the PA pulse pressure and RV stroke volume. Systolic PA pressure was estimated from the tricuspid regurgitation velocity by the modified Bernoulli equation. RV stroke volume was calculated using the pulmonary valve diameter measured in the parasternal short-axis view and the velocity-time integral of the PA Doppler flow [13]. In other reports, the right pulmonary arterial diameter change during the cardiac cycle was used to calculate PA capacitance [14]. However, these methods are complex and time-consuming, errors can easily occur, and they have low reproducibility. Previous reports that attempted echocardiographic estimation of PA capacitance all had limitations due to poor reproducibility and difficulty, because accurate quantitative assessment by two-dimensional echocardiography is hampered by the complex geometry and difficult depiction. Pulmonary arterial wall strain evaluated by speckle tacking method can be a candidate for evaluating PA distensibility and capacitance. However, in our preliminary experimental study, the tracking was insufficient and the reproducibility was quite low. Therefore, nongeometric methods to assess PA compliance and PA deformation should be explored. One such method, TDI, allows the quantitative assessment of longitudinal PA deformation.

To the best of our knowledge, this is the first application of pulmonary annular motion velocity obtained by TDI as a tool for PA capacitance assessment. We postulated that the PA longitudinal deformation reflects stroke volume, and that the pulmonary annular motion waveform shows PA capacitance.

In the present study, PA capacitance was represented by two parameters: sPAC and dPAC. There was a significant correlation between the two parameters, although sPAC was significantly higher than dPAC. The significant difference between the two parameters was assumed to be caused by the time phase difference during the cardiac cycle. The sPAC is the arterial capacitance from end-diastole to systole, whereas the dPAC is the capacitance from end-systole to diastole.

The peak velocities of the pulmonary annular s1′, s2′, e’, and a’ waves were significantly lower in the VSD group than in the Control group (Fig. 2), whereas the peak velocity of the tricuspid s’ wave was significantly higher in the VSD group than in the Control group (Table 1). This discrepancy between pulmonary and tricuspid annular motions might have resulted from the different RV contractility properties between RV inflow and outflow regional properties that originate from the RV geometry, including myocardial fiber orientation [15, 16]. Furthermore, we assumed that PA motion velocity might be affected by PA properties, including PA capacitance. The annular motions are presumed to be affected by RV performance and the adjacent structures [9]. The pulmonary annulus and tricuspid annulus are located adjacent to the PA and right atrium, respectively. Tricuspid annular motion would be affected by right atrial stiffness, contraction, and pressure. Pulmonary annular motion is assumed to be affected by PA properties, including arterial pressure, compliance, and stiffness. These conditions might contribute to the opposite results for the peak velocities of pulmonary and tricuspid annular motions in the VSD group.

The present data demonstrated that pulmonary annular motion parameters can be used to represent PA capacitance. On multiple regression analysis, PA capacitance independently affected the pulmonary annular motion waveform and parameters. Importantly, RVEF was not an independent variable that determined pulmonary annular motion. This is likely due to the fact that PA stroke volume is not correlated with RVEF because VSD shunt flow is directly expelled to the PA during systole in the VSD group. The present data indicate that pulmonary annular motion is determined not by RV contraction, but by PA deformation in children with VSD. Nevertheless, the present study demonstrated the significant negative correlation between the pulmonary annular s1′ wave and RVEDP in the VSD group. Furthermore, RVSP and RVEDP affect the peak velocity of the e’ wave. It would be useful to investigate the relationship between pulmonary annular motion velocity and RV performance or RV overload. Further studies are necessary to determine the various factors affecting pulmonary annular motion.

### Limitations

The sample cohort was relatively small, but TDI parameters were compared between patients and age-matched healthy individuals, and distinctive waveforms were found in the patient group. We postulated that PA expansion by the stroke volume and distensibility is similar in the radial and longitudinal directions. However, a directional difference might be present, which might generate systematic measurement error. The detailed characteristics of stiffness and elasticity in the PA radial and longitudinal directions should be elucidated in the future. Next, some degree of angulation between the Doppler beam and the true direction of longitudinal PA elasticity might exist. The long-axis view of the RVOT and PA was obtained, and the ultrasound beam was parallel to the direction of the pulmonary annular motions. We considered that this is the most appropriate cross-sectional view to observe the expansion and contraction of the PA.

A significant correlation between the s1′ wave and RVEDP was found. Furthermore, RVSP and RVEDP had effects on the peak velocity of the e’ wave in the VSD group. These indicate that RV performance also affects pulmonary annular motion. It is necessary to note that PA capacitance is not the only factor determining pulmonary annular motion. From another point of view, it would be meaningful to investigate the relationship between pulmonary annular motion velocity and RV performance or RV overload. Further studies are needed to determine the utility of pulmonary annular motion.

Lastly, although it was demonstrated that TDI-derived pulmonary annular motion parameters reflect PA capacitance, no attempt was made to determine the most useful parameter and derive the prediction formula to evaluate PA capacitance in the present study. The correlations found between pulmonary annular motion parameter and PA capacitance are relatively weak. Further studies of larger patient populations are needed to determine the most valuable parameter and the normal range of pulmonary annular motion for the evaluation of PA capacitance. Moreover, a further study with other disease populations would be necessary to establish the importance of the TDI-derived pulmonary annular motion waveform for the estimation of PA capacitance.

## Conclusions

Pulmonary annular TDI is a promising echocardiographic tool for evaluating PA capacitance in children with VSD. It can be a simple, rapid, reproducible, and highly distinctive method for evaluating PA capacitance.

## Additional file

Additional file 1: The pulmonary annular motion would be affected not only by RVOT function, but also by PA capacitance because the pulmonary annulus is located adjacent to the RVOT and PA. The deformation of arterial wall during systole and diastole occurs in the radial and longitudinal directions. The longitudinal pulmonary arterial deformation can reflect the vascular capacitance. (WMV 358 kb)

## Abbreviations

BSA: Body surface area
dPAC: Diastolic pulmonary arterial capacitance
DPAP: Diastolic pulmonary arterial pressure
HR: Heart rate
ICT: Isovolumic contraction time
IRT: Isovolumic relaxation time
LVEDD: Left ventricular end-diastolic dimension
LVEF: Left ventricular ejection fraction
LVFS: Left ventricular fractional shortening
MPAP: Mean pulmonary arterial pressure
PA: Pulmonary artery
PAH: Pulmonary arterial hypertension
PAPP: Pulmonary arterial pulse pressure
PASV: Pulmonary artery stroke volume
PVRi: Pulmonary vascular resistance indexed for body surface area
Qp/Qs: Pulmonary to systemic blood flow ratio
RV: Right ventricle
RVEDP: Right ventricular end-diastolic pressure
RVEF: Right ventricular ejection fraction
RVOT: Right ventricular outflow tract
RVSP: Right ventricular systolic pressure
sPAC: Systolic pulmonary arterial capacitance
SPAP: Systolic pulmonary arterial pressure
TDI: Tissue Doppler imaging
VSD: Ventricular septal defect

## References

[1] Rosenzweig EB, Widlitz AC, Barst RJ (2004). Pulmonary arterial hypertension in children. Pediatr Pulmonol.

[2] Raymond RJ, Hinderliter AL, Willis PW, Ralph D, Caldwell EJ, Williams W (2002). Echocardiographic predictors of adverse outcomes in primary pulmonary hypertension. J Am Coll Cardiol.

[3] Abman SH, Hansmann G, Archer SL, Ivy DD, Adatia I, Chung WK (2015). Pediatric Pulmonary Hypertension: Guidelines From the American Heart Association and American Thoracic Society. Circulation.

[4] McGregor M, Sniderman A (1985). On pulmonary vascular resistance: the need for more precise definition. Am J Cardiol.

[5] Mahapatra S, Nishimura RA, Sorajja P, Cha S, McGoon MD (2006). Relationship of pulmonary arterial capacitance and mortality in idiopathic pulmonary arterial hypertension. J Am Coll Cardiol.

[6] Mahapatra S, Nishimura RA, Oh JK, McGoon MD (2006). The prognostic value of pulmonary vascular capacitance determined by Doppler echocardiography in patients with pulmonary arterial hypertension. J Am Soc Echocardiogr.

[7] Sajan I, Manlhiot C, Reyes J, McCrindle BW, Humpl T, Friedberg MK (2011). Pulmonary arterial capacitance in children with idiopathic pulmonary arterial hypertension and pulmonary arterial hypertension associated with congenital heart disease: relation to pulmonary vascular resistance, exercise capacity, and survival. Am Heart J.

[8] Pellegrini P, Rossi A, Pasotti M, Raineri C, Cicoira M, Bonapace S (2014). Prognostic relevance of pulmonary arterial compliance in patients with chronic heart failure. Chest.

[9] Hayabuchi Y, Ono A, Kagami S (2016). Pulmonary annular motion velocity assessed using Doppler tissue imaging - Novel echocardiographic evaluation of right ventricular outflow tract function. Circ J.

[10] Reuben SR (1971). Compliance of the human pulmonary arterial system in disease. Circ Res.

[11] Stergiopulos N, Meister JJ, Westerhof N (1995). Evaluation of methods for estimation of total arterial compliance. Am J Physiol Heart Circ Physiol.

[12] Henriksen JH, Fuglsang S, Bendtsen F, Christensen E, Møller S (2001). Arterial compliance in patients with cirrhosis: stroke volume-pulse pressure ratio as simplified index. Am J Physiol Gastrointest Liver Physiol.

[13] Friedberg MK, Feinstein JA, Rosenthal DN (2007). Noninvasive assessment of pulmonary arterial capacitance by echocardiography. J Am Soc Echocardiogr.

[14] Dyer K, Lanning C, Das B, Lee PF, Ivy DD, Valdes-Cruz L (2006). Noninvasive Doppler tissue measurement of pulmonary artery compliance in children with pulmonary hypertension. J Am Soc Echocardiogr.

[15] Hayabuchi Y, Sakata M, Kagami S (2015). Right ventricular myocardial deformation patterns in children with congenital heart disease associated with right ventricular pressure overload. Eur Heart J Cardiovasc Imaging.

[16] Nielsen E, Smerup M, Agger P, Frandsen J, Ringgard S, Pedersen M (2009). Normal right ventricular three-dimensional architecture, as assessed with diffusion tensor magnetic resonance imaging, is preserved during experimentally induced right ventricular hypertrophy. Anat Rec.

